# Clinical trials and tribulations

**DOI:** 10.1038/s44319-024-00079-9

**Published:** 2024-02-05

**Authors:** Frank Gannon

**Affiliations:** QIMR Berghofer Medical Research Institute in Brisbane, Brisbane, QLD Australia

**Keywords:** Cancer, Economics, Law & Politics, Pharmacology & Drug Discovery

## Abstract

We need better post-approval monitoring and reporting to assess the efficiency of new cancer therapies in the real world beyond clinical trials.

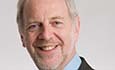

Randomized Clinical Trials (RCT) are the cornerstone of a lengthy and expensive process that ultimately leads to the approval of a new treatment for its use with patients. Basically, a RCT takes a number of volunteers who receive the new treatment and compare the outcome with those who received a placebo under the same conditions. If there is a statistically valid difference between the two arms of the trial in favour of the new approach, Bingo! The new drug now awaits a hopefully positive decision by the FDA, the EMA or other regulatory agency to give it a tick of approval. Skipping over the delicate question of costs (Gannon, 2023), patients can now look forward to a new lease on life thanks to the new treatment. Sharevalues spike for the company that owns the product. Hesitant investors become less shy about committing money to expansion plans required by the new addition to the portfolio. The benefits of investing in research and development are touted. The combination of hope and hype filter to the patient community. Tuned-in clinicians add the new treatment to their repertoire.

But, of the 161 drugs, approved by the FDA since 2017 for treating solid cancer in adults, only 35% were graded as “delivering substantial clinically meaningful benefit” on the European Society for Medical Oncology Scale, a standardized tool for assessing anticancer therapies. And an analysis of the benefits noted in RTCs for cancers shows that the increased overall survival rate is only 3.4 months and less than that, 2.9 months, for delaying signs of progression (Del Paggio et al, 2021). Several studies have repeatedly reported similar outcomes (Prasad, 2017; Davis et al, 2017; Grossman et al, 2020; Arciero et al, 2021; Falcone et al, 2022; Strand et al, 2023). Their results are different from the superlatives that are commonly used to highlight the latest “gamechanger” (Abola and Prasad, 2016). Indeed, the use of such language is not restricted to treatments that pass the RCT stage, they are even used for compounds that have not yet finished pre-clinical studies.

The problem arises from the fact that patients in the real world are challenged by their individual concerns about their illness, which increases their belief in the most optimistic outcome from the latest (available) treatment. The accumulated data since approval are seldomly highlighted, so the next wonderful drug is in reality less than it states on the tin - even if careful language ensures that no excessive promises are made. Faced with this dichotomy between promise and reality, a coalition called Common Sense Oncology have pointed out the multiple negative impacts of the current situation, including financial burdens, inequity of access, and the impact of side effects of treatments that may have only marginal benefits in terms of survival (Booth et al, 2023).

A closer look at the RTC process may therefore be needed. The lack of outstanding results in the clinics that use a treatment that came successfully through the RCT may be caused by the great care taken when enrolling patients in a clinical trial. Those with a secondary disease are excluded to avoid confounding the trial, which means that the volunteers are optimal candidates. The age profile also may not represent the generally older population of cancer patients. In the real world, people are not as ideal. During a RTC, patients are provided with the best care and monitoring during and after the treatment. In the real world, they may be moved from the clinic as soon as possible and end up in a situation, geographically or financially, where follow-up checks cannot take place as often as would be optimal.

In total, 80% of RTCs are funded now by industry (Abola and Prasad, 2016). That is a necessary part of the process as the public purse could not shoulder this investment. With the growth of industry as the driver of RTCs, it has become possible to increase the size of trials and hence the power of the statistical analysis that ultimately determines whether the new treatment is a success or not. When ever-bigger numbers are required to meet some statistical measure of success, it really means that the impact of the treatment on an individual may be a real possibility, but it is a small one. As my first post-doc supervisor said, “if you can only prove it by statistics, then it is not a real difference.” Of course, he knew that a result had to statistically sound but, for him, the impact of the experiment should be clear-cut. Those who watch presentations of pre-clinical and clinical data are drawn to the gap between the lines of the experimental and standard treatments. Converting that image into the number of months gained for a patient is difficult. Indeed, it is only after the real-world data accumulate that a real benefit can be seen. Or not.

Clinical trials are essential. New drugs are needed. Pharmaceutical companies do address societal needs. Investment is required to enable them to bring drugs to the clinic. Publicity and promotion is therefore part of the process to get the message not only to investors and the stock markets, but also to clinicians. The approval process carefully vets the data presented. But implementation takes place in the clinic under everyday conditions and that’s when the treatment faces its real trial in multiple settings with all of the human variations in the context of the illness. What seems to be missing is a feedback mechanism, with a similar level of publicity, when such data from the real world accumulate. That would allow more rational decisions to be made by patients, the funding systems that finance the new treatments, and the clinicians who use them.
